# Enhancing surgical efficiency: predicting same-day cancellations in urologic procedures

**DOI:** 10.1007/s00345-025-06155-6

**Published:** 2025-12-17

**Authors:** Pablo A. Suarez, Sudarshan Srirangapatanam, Lynn Leng, Mubarak M. Momodu, John Neuhaus, David B. Bayne

**Affiliations:** University of California, San Francisco, USA

**Keywords:** Social determinants of health, Health services accessibility, No-show patients, Health inequities, Urologic surgical procedures

## Abstract

**Purpose:**

Day-of-surgery (DOS) cancellations negatively impact healthcare efficiency and patient outcomes. Early identification of at-risk patients is essential for timely perioperative interventions. This study aims to develop and evaluate a predictive model for DOS cancellations in urologic surgeries using a comprehensive set of electronic health record (EHR) derived variables, including social determinants of health (SDOH).

**Methods:**

This retrospective cohort study examined adult patients scheduled for non-emergent urologic procedures at Zuckerberg San Francisco General Hospital (ZSFGH), a major safety-net institution. EHR data from January 2021 to June 2023 included demographics, social determinants of health, clinical history, and healthcare utilization. A lasso-optimized logistic regression model was used to predict DOS cancellations, with performance evaluated via area under the curve (AUC) of the receiver operating characteristic curve (ROC), accuracy, predictive values, sensitivity, specificity, and kappa at a 0.4 threshold to prioritize high-risk identification. Internal validation was conducted using cross-validation.

**Results:**

Of the 778 patients identified as undergoing elective urologic procedures, 136 (17.5%) had a DOS cancellation. Our prediction model consisted of 13 variables selected by optimizing the penalty (λ) of the lasso to achieve maximal accuracy. When evaluating the model on the validation data, it performed with an AUC of 0.80 [CI: 0.70–0.89], 83% accuracy, and a kappa of 0.20. The model also had 81% sensitivity and 55% specificity with a cut-off threshold of 0.4.

**Conclusion:**

This is the first study to integrate comprehensive SDOH data into a risk score model for predicting DOS cancellations. With an AUC of 0.80, the model offers a novel tool to help identify high-risk patients of DOS cancellation and guide preoperative interventions. However, further studies assessing the external validity of our model at other clinical sites are necessary.

**Supplementary Information:**

The online version contains supplementary material available at 10.1007/s00345-025-06155-6.

## Introduction

Day-of-surgery cancellations are defined as surgical cases that are canceled on the scheduled surgery date [1, 2]. It is a multifactorial problem documented worldwide with detrimental impacts across healthcare systems, reducing hospital productivity, increasing healthcare costs, and worsening delays in necessary patient treatment [3–5]. The loss of operational efficiency and financial burden from DOS cancellation can be attributed to wasted operating room time, underutilized staff, as well as the increased use of hospital resources to reschedule and repeat preoperative planning [6, 7]. As such, surgical cancellations pose an often overlooked risk to the quality and efficiency of healthcare delivery, which can be overcome with proper use of resources and operational interventions [8]. 

Prior literature suggests that 86% of elective surgery cancellations are preventable and that developing effective interventions requires a more comprehensive understanding of the reasons for cancellations. Hospital-related causes, particularly the unavailability of operating room time, were found to be the most common reason for same-day cancellations [9, 10]. On the other hand, patient absenteeism or no-shows are the most common patient-related factors and are found to vary across hospital systems and surgical specialties, with cancellation rates between 20–65% [9].

The application of statistical prediction models shows significant promise in predicting surgical cancellations based on medical and procedure-related factors [11, 12]. However, previous studies have failed to account for SDOH, which are widely documented in the literature as being important predictors of surgical cancellations [13–16]. In this work, we propose a predictive model using holistic EHR data and SDOH to predict DOS cancellations more accurately than prior models [17]. This may help early identification and targeted, cost-effective interventions to reduce DOS cancellations in urology.

## Materials and methods

### Study cohort and data collection

We conducted a retrospective study of patients with scheduled elective urologic procedures between January 2021 and June 2023 at ZSFGH, a safety-net hospital and level 1 trauma center. Our study cohort included all patients 18 years and older who had a scheduled elective urologic procedure. Patients who required an emergent procedure were excluded from the study to account for the dilution of signals from social factors in emergency settings. Patient data was abstracted from different sources within the EHR system, EPIC (Epic Systems Corporation, Verona, WI, USA) (see Table 1). Census-tract codes linked to patients’ home addresses were used to determine the area deprivation index (ADI), a measure of neighborhood socioeconomic disadvantage [18]. The study protocol was reviewed and approved by the UCSF Institutional Review Board (IRB 20-31513).

**Table 1:** Electronic health record (EHR) variables selected in this model

Data type	EPIC domains	No. of variables	Name of variables
Demographic data	Patient demographic information	7	Age, race, gender, sexual orientation, ethnicity, primary language
Social & behavioral data	Self-reported surveys; history & physical (H&P) provider notes	10	Education attainment, marital status, employment status, disability, wheelchair user, U.S. nativity, history of incarceration, history of medical respite, history of living in a single room occupancy (SRO), history of being unhoused
Clinical history	Clinical history; problem list	19	Congestive Heart Failure (CHF), hypertension, diabetes, chronic kidney disease (CKD), anxiety, mood disorders (depression/bipolar disorder), schizophrenia, post-traumatic stress disorder (PTSD), other mental health diagnosis, tobacco use disorder, substance use disorder, opioid use disorder, surgical procedure, surgical diagnosis, stent in place, nephrostomy tube in place, catheter in place, prior gynecologic procedure, prior urologic procedure
Healthcare access & utilization	Contact and care coordination information; EHR reports of clinical visits	11	Insurance type, MyChart activity status, primary contact on file, alternate contact on file, primary care provider on file, social worker on file, care coordinator on file, financial counselor on file, frequency of primary care provider (PCP) visits, frequency of Emergency Department (ED) visits, no show rate
Census tract data	Public geocoded data linked to patients’ home address	1	State-level area of deprivation (ADI) scores

### Primary outcome definition

The primary outcome was a binary indicator of DOS cancellation, which was defined as any scheduled procedure cancelled on the same day of surgery. Patients who experienced at least one DOS cancellation during the study period were classified as having the outcome. In contrast, patients who had multiple scheduled surgeries but completed all of them as planned were classified as not having the outcome and assigned to the procedure performed group.

### Candidate predictors selection

We selected candidate predictors a priori based on existing literature, prioritizing variables with established associations to appointment attendance, no-shows, or service cancellations. Other variables were included based on established SDOH domains. The definitions of candidate predictors are presented in Table 1. Due to inconsistent reporting of SDOH in the EHR, we used targeted search terms guided by clinical expertise to extract data from social history, clinical notes, and patient-reported information (Online Resource 1).

All 48 candidate predictors were collected without missing data. The dataset was randomly split into 70% training and 30% validation sets. A random oversampling technique was used to prevent model overfitting due to the imbalance between performed versus DOS cancellation, involving randomly replicating observations in the minority class until both classes are sufficiently balanced [19]. 

A logistic regression model with a lasso regularization technique was fitted to optimize the predictor selection of only those truly associated with the outcome and to reduce collinearity [20]. From a grid of 48 penalties, the optimal penalty tuning parameter (λ) was determined by optimizing for maximal prediction accuracy (Online Resource 2).

### Prediction model performance evaluation and validation

The performance of the prediction model generated was assessed through multiple evaluation methods. The AUC of the ROC curve was used to measure discrimination ability. A kappa statistic was calculated to determine agreement between the predicted and actual classifications while accounting for agreement occurring by chance [21]. 

Where Po ​is the proportion of instances where the model and the actual outcomes are concordant, and Pe is the expected agreement by chance computed from the marginal probabilities of each outcome.

The predictive accuracy of the model was defined as the proportion of all correct predictions (both DOS cancellation and performed procedures) out of the total number of predictions, and it was calculated as shown. Additionally, we evaluated the model’s validity by calculating negative predictive value (NPV), positive predictive value (PPV), sensitivity, and specificity according to the following equations (Online Resource 3).

The true positives (TP) are the number of correctly identified DOS cancellations predicted by our model, and the false positives (FP) are the number of incorrectly predicted DOS cancellations when the procedures were in fact performed. On the other hand, the true negatives (TN) are the number of procedures correctly predicted to be performed, and the false negatives (FN) are the number of procedures that were predicted to be performed when in fact they were DOS cancellations. These metrics were calculated at an optimal threshold selected via parameter optimization (Online Resource 3).

Model validation was performed by dividing the data into a training set (70%) for model development and a test set (30%) for performance evaluation. The validity metrics mentioned above were applied to the test set. The cross-validation was performed in three different sets of training and validation sets to further demonstrate our model’s internal validity. All statistical analyses were conducted using Stata version 18.0 (Stata Corp, College Station, TX). Results were reported with a 95% confidence interval, and statistical significance was defined as *p* < 0.05.

## Results

### Lasso-selected predictors of DOS cancellations

The DOS cancellation rate in our cohort was 17.5% (131 out of 778). Amongst the reasons for procedure cancellations were no-shows (45.6%), day of surgery changes to the OR schedule (14.0%), patient was not medically cleared (13.2%), COVID-related cancellations (5.9%), surgery no longer indicated (2.9%), and other reasons (18.4%).

**Fig. 1:**
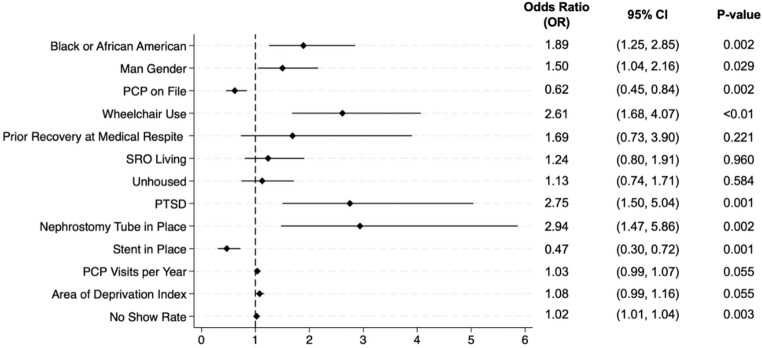
Forest plot of LASSO-selected predictors

We identified 48 candidate predictors a priori from the literature as detailed above. At the λ value of 0.03 optimized for maximal accuracy (Online Resource 2), 13 variables were selected and found to be moderate to strong predictors of DOS cancellations using multivariable logistic regression. Estimated odds ratios (OR) and associated 95% confidence intervals (CI) for Black or African American (BAA) race were OR = 1.89 and CI= [1.25, 2.85], and for male gender were OR = 1.50 and CI= [1.04, 2.16] (see Fig. 1). Factors related to healthcare coordination, access, and utilization that had a strong association with DOS cancellations included primary care provider (PCP) on file (OR = 0.62; CI= [0.45, 0.84]), and no-show rates (OR = 1.02; CI= [1.01, 1.04]) (see Fig. 1). Surgical and medical history associated with DOS cancellations were post-traumatic stress disorder (PTSD) (OR = 2.75; CI= [1.50, 5.04]), having a nephrostomy tube (OR = 2.94; CI= [1.47, 5.86]), and a stent in place prior to the scheduled surgery (OR = 0.47; CI= [0.30, 0.72]) (see Fig. 1). Wheelchair use was the only significant socio-behavioral factor associated with DOS cancellation (OR = 2.6; CI= [1.68, 4.07]). Number of PCP visits per year (OR = 1.03; CI= [0.99–1.07] and ADI (OR = 1.08; CI= [0.99–1.16] were marginally significant predictors of DOS cancellations (see Fig. 1).

Logistic regression using variables identified via Lasso selection optimization technique. BAA race, man gender, PCP on file, wheelchair use, PTSD, nephrostomy tube in place, stent in place, and no-show rate were significant predictors of DOS cancellations (*p* < 0.01).

### Performance of the DOS cancellation prediction model

**Fig. 2:**
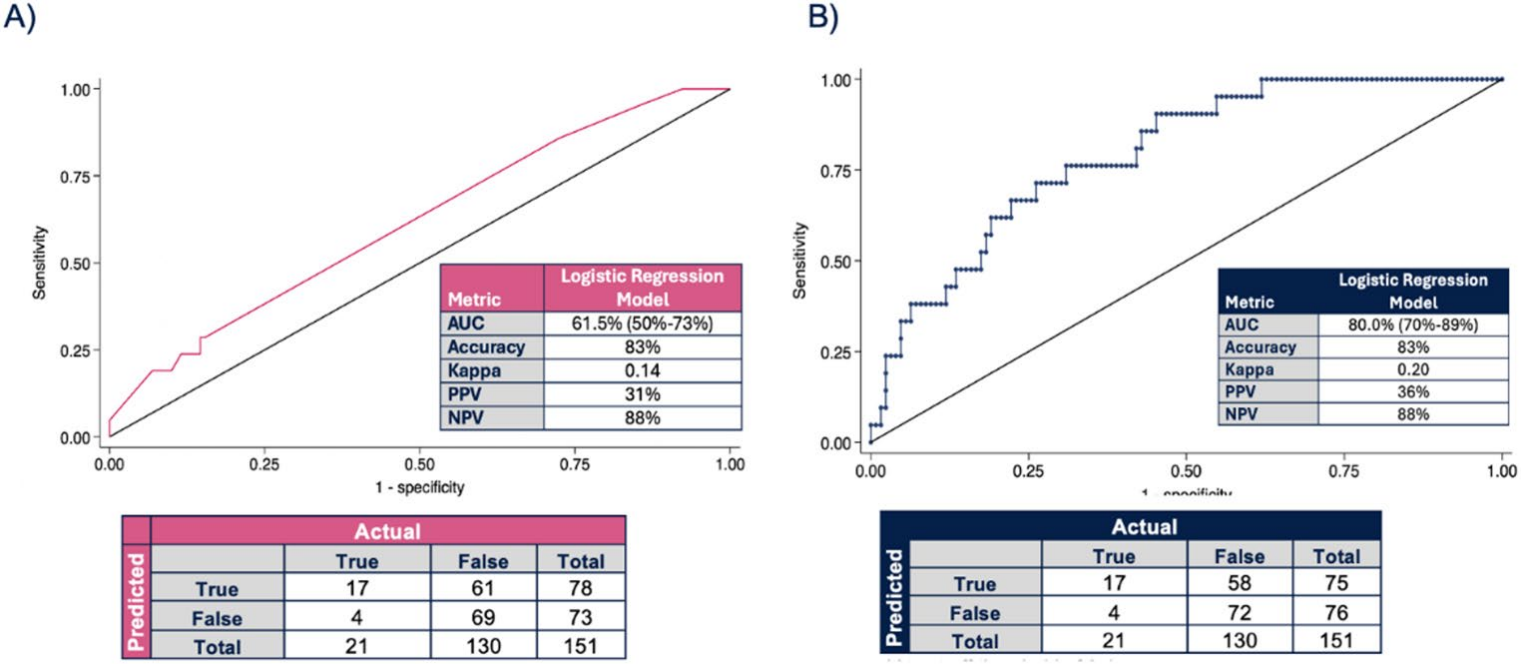
Receiver Operating Characteristic (ROC) curves and contingency matrices comparing model performance with and without comprehensive social determinants of health (SDOH) factors. A. Simple Model using only demographic and clinical variables. B. Comprehensive Model incorporating using demographic, clinical, social/behavioral, healthcare access/utilization, and census tract variables.

A basic model, non-inclusive of SDOH variable, had an AUC of 62% and a predictive accuracy of 83% (see Fig. 2A). When including the SDOH factors, along with the rest of the lasso-selected variables, the AUC increased to 80% and maintained a similar predictive accuracy of 83% (see Fig. 2B). The kappa statistic was 0.20, corresponding to a fair agreement according to Cohen’s range (see Fig. 2B) [20]. At an optimal binary predictive threshold of 0.4, the model accurately predicted DOS cancellation with a sensitivity of 81% and specificity of 55% (see Fig. 2B; Online Resource 4).

## Discussion

Given that 71% of DOS cancellations are preventable, this study proposes a logistic regression model to predict possible DOS cancellations using EHR patient data from multiple domains [4]. Our logistic model, which included 13 patient-specific variables, demonstrated high predictive capacity for DOS cancellations, achieving an AUC of 0.80 and an accuracy rate of 83%. Using our model, we can identify DOS cancellations with a sensitivity of 81% by optimizing our binary prediction threshold at 0.4. This tool may enable timely and potentially cost-effective interventions for high-risk patients, leading to improved healthcare productivity and greater access to urologic care.

With the development of prediction models, there has been an increased interest in leveraging these tools to predict clinical no-shows and cancellations. Recent work has shown that operating room and clinic cancellations can be predicted with an AUC greater than 70% when integrating patient-specific socioeconomic, clinical, and environmental factors [13–16]. Wongtangman et al. developed an instrument to determine the incidence of case cancellations within 24 h for elective surgeries using perioperative data and procedural factors [13]. However, this study was constrained by a narrow scope of EHR-derived behavioral and socioeconomic data. Our study shows that incorporating diverse SDOH proxies from the EHR improves DOS cancellation prediction, raising AUC to 80% (vs. 70%) with an accuracy of 83%.^13^

Among elective surgeries, urology has one of the highest same-day cancellation rates compared to other subspecialties, reaching 9.53%, while general surgery has a lower cancellation rate of 8.2% [22]. Although over half of these cancellations are considered avoidable, no prior study has explored using SDOH to predict day-of-surgery cancellations in urologic procedures. The only other prediction model for DOS cancellation in urology was conducted in China and was limited to OR scheduling data, such as surgeon, surgical category, and whether this was the first performed surgery of the day [16]. In contrast, our study demonstrates that the inclusion of SDOH factors raises the AUC from 62% to 80%, highlighting the importance of social risk assessment in predicting DOS cancellation.

Even though equal access to healthcare facilities is required by law under the Americans with Disabilities Act (1990), people with disabilities continue to face unequal access to care due to mobility issues, inaccessible equipment, and inadequate policies [23]. We demonstrate that wheelchair use, as a proxy for mobility limitation and transportation to care barriers, can strongly predict DOS cancellation. Our study also demonstrates that patients at risk of housing insecurity, as indicated by prior history of medical respite recovery, single room occupancy (SRO) or low-income subsidized housing, and being unhoused, are selected predictors for DOS cancellations. This supports prior studies that indicate that housing instability is associated with unmet healthcare needs and worse postoperative outcomes [24, 25]. 

Healthcare accessibility and utilization factors, such as the presence of a PCP on file, the number of annual PCP visits, and no-show rates, had a noted effect on DOS cancellation. No-show rates are an EHR-reported metric previously linked with increased risk of DOS cancellation [13, 26]. Conversely, the presence of a primary care doctor or referring specialist is linked to lower no-show rates for outpatient procedures [27]. Prior history of surgery as a proxy of access to care has also been found to be protective against DOS cancellation [13]. In our cohort, having a ureteral stent before surgery reduced the odds of day-of-surgery cancellations by 53%, likely due to better access to surgical care or the need for definitive treatment after stent exchange. Conversely, as previously reported, nephrostomy tubes were linked to higher cancellation risk, reflecting poor follow-up compliance [28]. 

Racial minority status, particularly BAA race status, is a well-documented non-modifiable predictor of DOS cancellations for patient absenteeism across subspecialties [29, 30]. This work builds on existing evidence showing that patients who identify as BAA face greater challenges in accessing surgery, contributing to healthcare disparities such as delays in necessary operations and poorer postoperative outcomes for both benign and malignant urologic conditions [31, 32]. Structural barriers disproportionately impact access to care for racial minorities, and as such we adjusted for ADI, a common socioeconomic risk measure reflecting neighborhood healthcare infrastructure. Even after this adjustment, BAA race remained a significant predictor, underscoring the need to explore structural causes behind racial disparities in DOS cancellations.

Non-attendance at clinical appointments is a marker of mental health diagnosis in young adults [33]. There has been a strong association reported between a diagnosis of anxiety and DOS cancellation [13]. While we did not find anxiety to be associated with DOS in our model, we found PTSD to more than double the risk of DOS cancellation for urologic procedures in our study cohort. It is possible that prior studies may have conflated PTSD with symptoms of anxiety, as both conditions are highly comorbid and share similar neurobiological features. We are the first study to report that PTSD increases the risk of surgical cancellation and signal the importance of mental health screening to optimize surgical attendance.

At our safety-net hospital, the DOS cancellation rate was notably high, at approximately 18%. Like many safety-net institutions, ZSFGH serves communities with elevated social vulnerability. Our patient population faces higher levels of social risk factors, including lack of insurance, minority status, and economic hardship, which may contribute to the increased rate of DOS cancellations. Based on these findings, we propose this model can either replace or serve as an adjunct to social screening tool. By using this information to risk-stratify patients according to unmet social needs, healthcare providers can more effectively deliver early preoperative counseling, social support services, and more frequent communication reminders prior to surgery as suggested by the work of Hovlid et al. [34] Addressing both the medical and social aspects of patients’ lives in conjunction may help reduce surgical cancellation rates, particularly among underserved populations.

This study is not exempt from limitations. The generalizability of our study is limited as this was a single-center urology-focused study at a county hospital serving a uniquely diverse and vulnerable population. A very important limitation of this work is the absence of data on operational factors, such as timing of surgery, staffing, and availability and access of same-day testing or imaging for adequate optimization of surgical candidates preoperatively. There was also a significant lack of standardized reporting of education, employment, and immigration status in our EHR system, resulting in missing data. In our study, missing data were remedied by a systematic manual abstraction using the search bar of the EHR. This approach is susceptible to human error, misclassification, and personal biases during data abstraction. In addition, manual chart review for SDOH proxies may pose a significant barrier in the replicability of this study, particularly in resource-limited settings. As a result, our work encourages the implementation of a standardized social screening to facilitate the collection of this information given its significant impact on DOS cancellations 

Despite limitations, our study presents a novel prediction model using patient-specific factors that identifies individuals at high risk of DOS cancellations with 81% sensitivity and 55% specificity. In a validation set of 150 patients, screening 50% identified 81% of cancellations. If 75% effective in the full cohort, this could prevent 74 of 131 cancellations, reducing DOS cancellations by 10%. This targeted approach can improve operating room efficiency by reducing the need for universal screening. In addition, future modeling that includes operational factors is needed for improve our ability to successfully predict surgical cancellations accounting for external factors, such as timing of surgery, staffing, and operational resource. Further multicenter studies are needed to confirm the effectiveness and external validity of these models in urologic practice.

## Conclusion

Given urology’s high rate of preventable DOS cancellations, we used a logistic regression model to predict cancellations based on EHR-derived data. Our model demonstrates strong predictive performance (AUC = 80.0%) and represents a novel screening tool that could be used in future quality improvement initiatives to reduce same-day surgical cancellations and promote equitable access to urologic care.

## Supplementary Information

Below is the link to the electronic supplementary material.

Supplementary Material 1

Supplementary Material 2

## Data Availability

The data underlying this study contain protected health information and cannot be publicly shared due to institutional and federal privacy regulations. De-identified datasets may be made available from the corresponding author upon reasonable request and with approval from the Institutional Review Board.
