# Supplementary material for: Enhancing surgical efficiency: predicting same-day cancellations in urologic procedures
Source: World J Urol. 2025 Dec 17;44(1):48. doi: 10.1007/s00345-025-06155-6 (PMC12712017; doi:10.1007/s00345-025-06155-6)
Supplement: Supplementary file 1 — Supplementary Material 1 [file 345_2025_6155_MOESM1_ESM.docx]

### **Supplementary Information (SI)**

#### **Online Resource 1:** Systematic Approach to Manual Data Abstraction for missing SDOH Variables

**​​**

| **SDHO Domains^a^** | **Variable** | **Description of Systematic Manual Data Abstraction** |
| --- | --- | --- |
| **Education Access & Quality** | **Education Attainment** | 1. Select “Social documentation” on history tab 2. Using the search bar function, search for “education” or “school” |
| **Economic Stability** | **Employment** | 1. Select “Social documentation” on history tab 2. Select patient demographics and find information under” occupation 3. Using the search bar, search for “occupation,” “employment,” and “work” |
| **Social & Community Context** | **U.S Nativity** | 1. Select “Social documentation” on history tab 2. Using the search bar, search for “born in,” “originated from,” and “immigrant” |
|  | **Hx of Incarceration** | 1. Using the search bar, search for “incarceration,” “in jail,” and “in prison” |
| **Neighborhood & Built Environment** | **Wheelchair Bound** | 1. Using the search bar, search for “wheelchair” and check for chronic dependency. If only temporary use, then it does not meet criteria |
|  | **Hx of SRO Living** | 1. Using the search bar, search for “SRO” |
|  | **Hx of being unhoused** | 1. Using the search bar, search for “unhoused” and “homeless: |
| **Healthcare Access & Quality** | **Opioid Use History** | 1. Prior history of opioid use on medical history 2. Using the search bar, search for “ward 93” (opiate treatment outpatient program) |
|  | **Hx of Medical Respite** | 1. Using the search bar, search for “medical respite” |

^a^ Five Domains of Social Determinants of Health established by the U.S Department of Health and Human services.

####

#### **
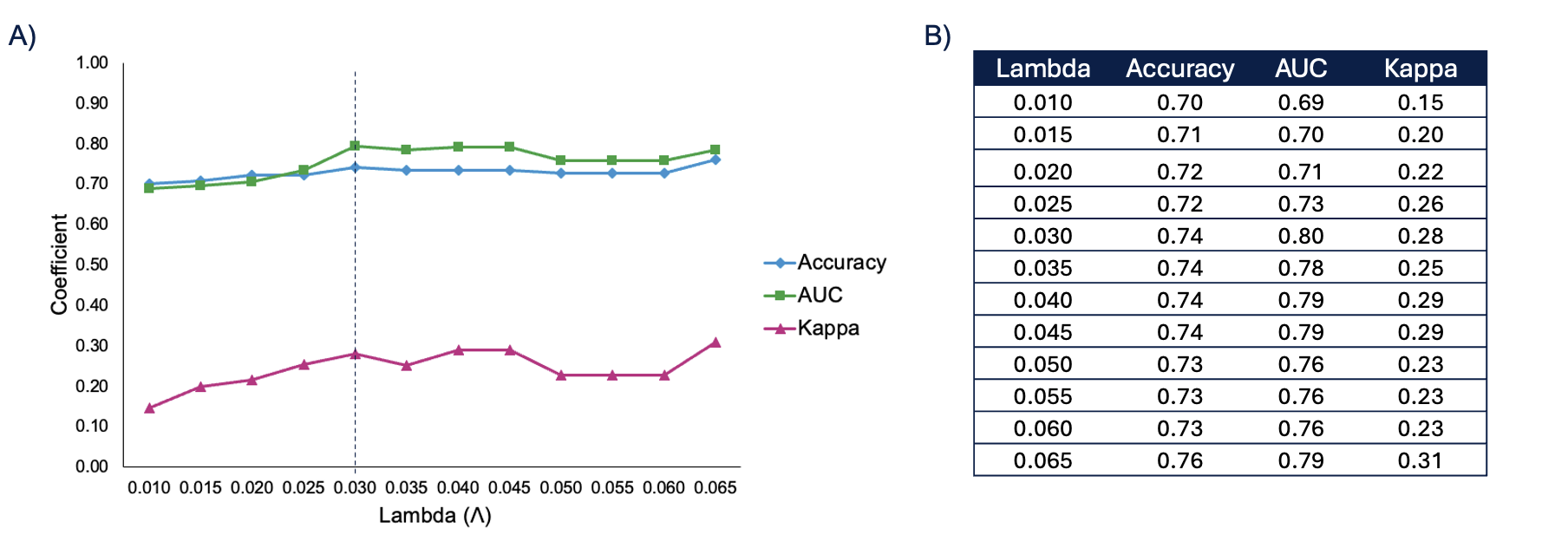
Online Resource 2:** Tuning parameter of lasso λ value

#### **Online Resource 3:** Critical threshold hyper-tuning selected at 0.60

**
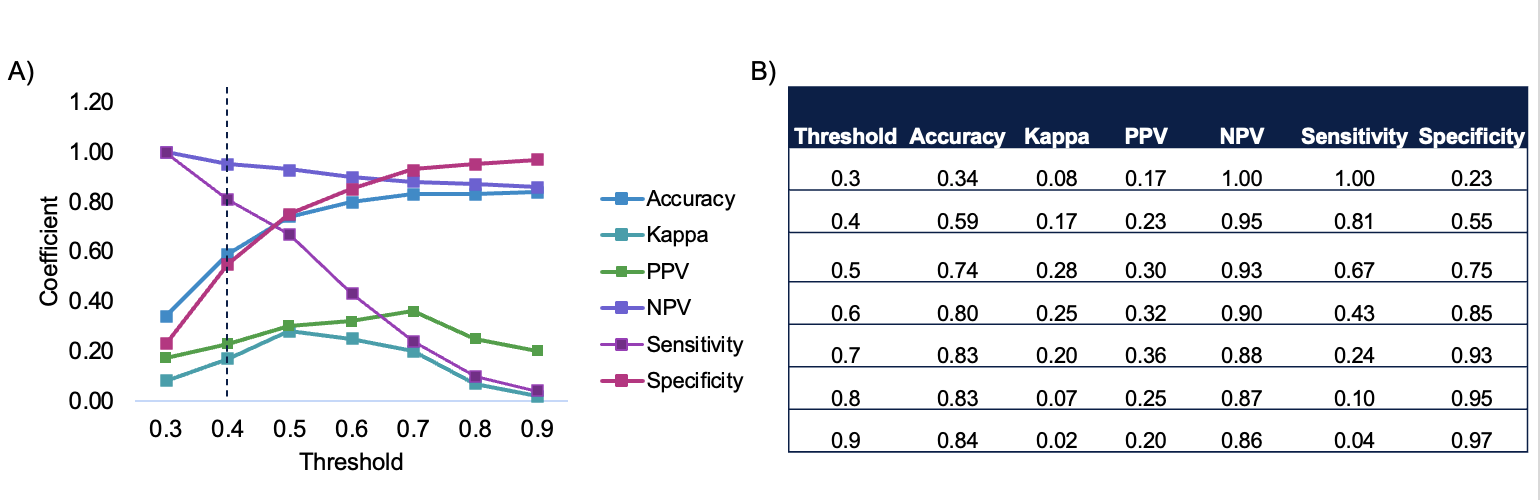
**
